# Mechanisms Driving Galling Success in a Fragmented Landscape: Synergy of Habitat and Top-Down Factors along Temperate Forest Edges

**DOI:** 10.1371/journal.pone.0157448

**Published:** 2016-06-16

**Authors:** Nina-S. Kelch, Frederico S. Neves, G. Wilson Fernandes, Rainer Wirth

**Affiliations:** 1 Plant Ecology & Systematics, University of Kaiserslautern, Kaiserslautern, Germany; 2 Departamento de Biologia Geral, Instituto de Ciéncias Biológicas, Universidade Federal de Minas Gerais—UFMG, Belo Horizonte, Minas Gerais, Brazil; 3 Department of Biology, Stanford University, Stanford, California, United States of America; University of Fribourg, SWITZERLAND

## Abstract

Edge effects play key roles in the anthropogenic transformation of forested ecosystems and their biota, and are therefore a prime field of contemporary fragmentation research. We present the first empirical study to address edge effects on the population level of a widespread galling herbivore in a temperate deciduous forest. By analyzing edge effects on abundance and trophic interactions of beech gall midge (*Mikiola fagi Htg*.*)*, we found 30% higher gall abundance in the edge habitat as well as lower mortality rates due to decreased top-down control, especially by parasitoids. Two GLM models with similar explanatory power (58%) identified habitat specific traits (such as canopy closure and altitude) and parasitism as the best predictors of gall abundance. Further analyses revealed a crucial influence of light exposure (46%) on top-down control by the parasitoid complex. Guided by a conceptual framework synthesizing the key factors driving gall density, we conclude that forest edge proliferation of *M*. *fagi* is due to a complex interplay of abiotic changes and trophic control mechanisms. Most prominently, it is caused by the microclimatic regime in forest edges, acting alone or in synergistic concert with top-down pressure by parasitoids. Contrary to the prevailing notion that specialists are edge-sensitive, this turns *M*. *fagi* into a winner species in fragmented temperate beech forests. In view of the increasing proportion of edge habitats and the documented benefits from edge microclimate, we call for investigations exploring the pest status of this galling insect and the modulators of its biological control.

## Introduction

Forest fragmentation is ranked amongst the leading mechanism behind the current global biodiversity crisis and belongs to the most pervasive impacts of human land use [1,2]. As a consequence of ongoing fragmentation, human-modified landscapes are now faced with an escalating proportion of edge habitats at the expense of continuous forest interior [3,4]. The resulting edge effects play key roles in the anthropogenic transformation of forested ecosystems and their biota, and are therefore a prime field of contemporary fragmentation research [5–8]. Currently, edge-induced alterations of species interactions receive particular close attention and there is considerable research effort underway to evaluate both the nature and intensity of these changes as well as cascading ecological consequences throughout a web of relationships. In fact, the disruption of trophic interactions has been repeatedly identified as a key mechanism involved in edge-mediated loss of ecosystem function or biodiversity [9–11] yet the understanding of the underlying causal processes is often sketchy. Among all trophic relationships, insect herbivory is widely recognized as one of the key processes shaping plant communities and influencing ecosystem function [12,13]. A general trend observed within the edge literature is a positive response of herbivores to forest edges. Especially generalist herbivores benefit from favorable micro-environmental conditions, increased food quantity and quality, including higher nutrition content and less defense capacity of plant tissue [8]. They may also profit from a disrupted top-down regulation, yet this is less well documented [11]. However, while several studies showed a clear profit of generalist herbivores to edge-driven changes, empirical evidence on specialized herbivores is less clear [8].

Due to their sessile life style and their high degree of host-specificity, gallers are regarded as particularly promising target taxa to compare the influence of different factors in a spatial context, and link cause to effect [14]. Limited research exists, however, investigating edge effects on gallers, most of it concentrating on gall diversity rather than species specific responses [15–18]. In fact, to our knowledge there is no study available addressing anthropogenic edge effect on gall species at the population level (see [16] for a study across a natural ecotone).

Here we used the galling insect–host plant system *Fagus sylvatica* L. (European beech) and *Mikiola fagi* Htg. (beech gall midge; Diptera: Cecidomyiidae). This species pair provides an excellent model system for our work for the following reasons. The beech gall midge induces egg-shaped, unilarval galls [19], which are shed (before leaf fall) for overwintering in the leaf litter. It is the most common and important herbivore species associated with beech [20,21] and there is considerable knowledge available about factors regulating its population density, ranging from (i) top-down control through a parasitoid complex [20, 22], and gall predation (e.g., by birds [23]), to (ii) bottom-up induced plant defense, called hypersensitivity reaction (HR) by the host tree *F*. *sylvatica* [24], and (iii) environmental conditions, such as increased gall infestation rates following high light exposure [25]. On the other hand, *F*. *sylvatica* is among the most abundant and economically important broad-leaved trees in the northern hemisphere and a key element of the European beech forest [26]—a unique European ecosystem with global conservation value as reflected by its UNESCO world natural heritage status [27]. The relevance of fragmentation-related studies on these forests is emphasized by the remarkable dominance and shade casting ability of beech [28]. As a result, the contrast between beech forest interior and edge is expected to be more abrupt compared to structurally more heterogeneous forest types.

In this paper we aimed at a detailed assessment of edge impacts and underlying mechanisms on the beech–gall midge system by collecting habitat-, resource-, and top-down-related data from the individual tree to the stand level and sampling over 10.000 galls on more than 300 individual beech trees across a fragmented beech forest landscape. We hypothesized that at the forest edge *M*. *fagi* galls (1) are more abundant because of their documented preference for light-exposed sites and (2) benefit from disrupted top-down control exerted by parasitoids, as implied by the trophic-level hypothesis of island biogeography (i.e. populations of higher trophic levels are more likely to become extinct under fragmentation conditions [29]) and empirical evidence for edge sensitivity of parasitoids [8]. To identify possible links and interactions between gall density, characteristic habitat traits, and biotic factors, we adopted several statistical models and, finally, generated a conceptual model that synthesizes key factors driving gall density across fragmented beech forest landscapes.

## Materials and Methods

### Study region and target species

**Fig 1:**
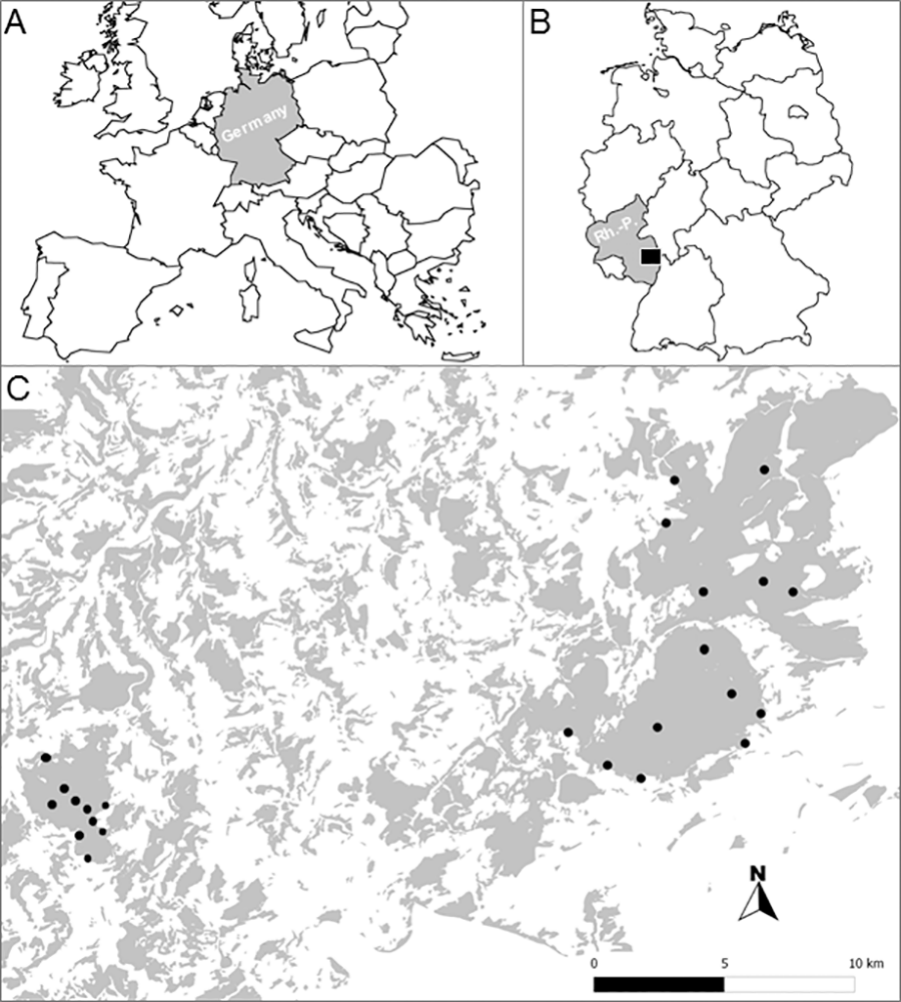
**Maps showing the situation of the Northern Palatinate highlands, with respect to Central Europe (A) and SW Germany (B), indicated as black rectangle in the state of Rhineland-Palatinate (Rh.-P.)**. The study landscape (C) shows forest fragments (grey polygons) embedded in a matrix of agricultural land uses (white) and 24 randomly established study plots (black dots), along forest edges and in forest interiors within continuous control forests (> 1,000 ha).

The study was conducted in the Northern Palatinate Highlands (Fig 1, a low mountain range (250–687 m ASL) in SW Germany (federal state of Rhineland-Palatinate). No endangered or protected species were involved. Permission to conduct the study in the respective forest areas was given by the Rhineland-Palatinate Ministry for Environment, Agriculture, Nutrition, Viticulture and Forestry. Carboniferous and Permian bedrock and sediments as well as Rotliegend volcanic rocks give distinction to the undulating landscape [30]. The climate is temperate and under oceanic influence with average temperatures ranging from of 8–9°C and mean annual precipitation from 550–800 mm [31].

The region is a human-modified landscape consisting of a mosaic of land uses and covers. Forests are deciduous, broad-leaved woodlands (Carpino-Fagetalia mixed forests) and have been subjected to extensive deforestation dating back to the Middle Ages. Land use is concentrated in sand and siltstone-dominated valleys, while the agriculturally less valuable igneous hilltops remain mostly forested. This fragmentation history has resulted in a landscape of hyper-fragmented forests, embedded in a matrix of pastures, cultivated fields (mostly root crops, cereals, malting barley and rape), and meadow orchards. Total forest cover of the study landscape (1,010 km²) amounts to 32%; as throughout Germany it remained largely stable across the past decades [32]. In order to exclude fragmentation effects other than edge effects, we selected the three largest forest remnants (under state property) nearby the towns of Wolfstein (49°35′3″N, 7°36′22″E; 1049.37 ha) and Kirchheimbolanden (49°40′01′N, 7°58′48″E; 5616.2 ha), and the forest area around the Donnersberg (49°37′31″N, 7°54′53″E; 3511.49 ha). The age of forest edges varied from about 40 to 130 years, most of them showing a smooth and stable transition from forest interior to the surrounding matrix. Silvicultural management frequently promotes beech (*Fagus sylvatica*), which reached higher dominance scores (59.3 ± 35.8%) than any other tree species (e.g., *Carpinus betulus* and *Quercus petrae*) in the interior of the studied forests. On the other hand the dominance of *F*. *sylvatica* was reduced (35.3 ± 23.6%) along the forest edge (Kelch N. unpublished diploma thesis).

The high abundance of beech and the widespread occurrence of its dominant herbivore *M*. *fagi* made the Northern Palatinate Highlands an interesting opportunity to survey temperate forest fragmentation. The conspicuous galls are easy to identify/count and provide simple access to a population-related data. The conical, glabrous galls become visible around the first two weeks of May and complete the growth by the end of September, beginning of October.

### Sampling design and scope

From July through September 2011, we inspected gall abundance in the understorey of 12 “forest edge plots” and 12 “forest interior plots” (control plots). All following analyses that explore factors driving gall density are based on data from the understorey stratum. However, to allow for comparisons with previous *M*. *fagi* studies, we also conducted surveys at the stand level. The term “stand level” refers to the collection of leaf litter and galls from the forest floor at the end of the season and therefore represents data from understorey and canopy together. Such extension from the understorey to the whole canopy may influence the results due to different proportions of sun leaves.

Study plots were established in 2008 as permanent plots based on aerial photographs of the Northern Palatinate Highlands (kindly provided by the German Research Institute for Forest Ecology and Forestry, Trippstadt) and extensive on-site surveys to evaluate plot placement with respect to current or planned management practices (e.g. large silvicultural clearings) and minimum edge proximity of forest interior plots (Fig 1). Each rectangular plot covered an area of 0.1 ha (50 m x 20 m). While edge plots were established parallel to the forest edge, the orientation of control plots in the forest center was arranged randomly at a distance of >100 m to the forest edge [7]. Plot elevation ranged from 250 to 687 m; interplot distance ranged from no less than 500 m between adjacent plots to as far as 30 km across the landscape (see S1 Table for additional plot characteristics). Since the spatial configuration of fragmentation in the study landscape is characterized by a higher location of forest remnants on hilltops, the control plots were usually located at higher altidudes than edge plots.

### Gall density survey

We examined gall density in the understorey by placing a sampling frame (0.24 m²) onto a randomly chosen branch of each tree per plot (using dice-generated numbers assigned to consecutively clockwise numbered branches). Ten leaves were taken out of that frame to count galls per leaf and to relate them to their respective leaf area (LI-3100 area meter, LI-COR). Gall density per plot was expressed as number of galls per cm² leaf area.

For the stand-level assessment, leaf litter was collected in five edge and five control plots from November to December 2011. To cover small-scale variation in leaf litter around the trees, three 0.25 m² litter samples per tree (i.e. 0.75 m²) were collected at half the distance of the three longest crown radii. Ten complete leaves (this year's leaf fall) per subsample were used for leaf area determination. Gall counts included distinct leaf marks (indicating previous gall location) and galls persisting on the leaf.

### Explanatory variables

To cover a range of habitat traits and environmental variables with potential impacts on the interactions involved in our galling insect–host plant system, we considered habitat type (forest edge vs. forest interior), vegetation density (number of stems per plot) and canopy closure as independent explanatory variables for variation in gall abundance (hereafter gall density). All three variables are linked to light climate (radiation interception and concomitant microclimatic changes such as temperature and moisture) and light exposition seems to be highly correlated with gall density [25]. Canopy closure as a measure of relative light intensity was quantified using a spherical densitometer leveled at the position of the sampling frame [33]. In addition, we chose altitude (measured with a Garmin GPS, eTrex H) as an independent variable, because the range of plot altitudes reached more than 400 m. Altitude involves climatic changes such as difference in temperature regimes, which could relate to the harsh environment hypothesis, predicting that gall inducing insects in general are associated with harsh ecophysiological conditions [34].

Finally, we examined the importance of host plant availability as a potential driver of gall density. Following the resource concentration hypothesis (sensu [35]), the occurrence of beech should have an impact on the abundance of monophagous *M*. *fagi*. To test this, we related gall density to the dominance of *F*. *sylvatica*, expressed as the basal area (BA) contribution of beech relative to the total BA of all tree species per plot. The BA (BA = π * r²) was obtained from measurements of DBH (diameter at breast height).

### Quantifying top-down control of *M*. *fagi*

In order to address higher trophic interactions as potential key mechanism behind the variation of gall density, we focused on mortality caused by the parasitoid complex as well as other factors. To assess mortality rates, all collected galls were opened and the gall contents were recorded as frequency counts. We distinguished five different mortality causes: “ectoparasitoid”, “endoparasitoid”, “predation”,“fungus” and “unknown”. Ectoparasitoids can unmistakably be discriminated from endoparasitoids, since the former lie next to the *M*. *fagi* larvae and suck out their body fluids. Endoparasitoids on the other hand live inside *M*. *fagi*, turning its skin into a dry, brown cocoon-like cover. In both cases the gall itself had no macroscopic marks or damages. In contrast, birds and caterpillars [22] harm the gall from the outside. Due to the damaged appearance those galls could easily be assigned to the “predation” category, which therefore includes predation on the gall tissue (indirect harm) and/or galling larvae [36]. Fungal hyphae inside the gall were regarded as gall content/mortality cause, although it was frequently impossible to distinguish primary from secondary fungal infections. Whenever the gall was empty, but no *M*. *fagi* larvae or other organism was found, we defined the mortality cause as “unknown”. Each group rate was calculated from the numerical proportion of galls per mortality group and total number of examined galls. Additionally, we summed up all mortality groups to obtain total mortality rate. To give an overview of survival success of *M*. *fagi* at the end of the season, before overwintering starts, we listed gall contents including, ecto- and endoparasitoids, fungus and inquilines (other larvae than *M*. *fagi* or adult arthropods, e.g. ants). Empty galls could be “open + empty” (intact gall, but showing no membrane at the opening where the gall was connected to the leaf) or “damaged + empty”.

### Data analysis

Differences in gall abundance between forest edge and forest interior at two spacial scales (understorey and stand level) were examined using generalized linear models (GLMs) [37]. GLMs were also applied to identify and quantify possible connections between gall density, resource availability and habitat types. In theses models gall density in the understorey was considered the response variable. As explanatory variables we used vegetation density, dominance of *F*. *sylvatica*, habitat type (forest edge versus interior) as well as the interactions between habitat type and both vegetation density and *F*. *sylvatica* dominance. The minimal model was constructed by removing nonsignificant explanatory variables (P > 0.05) from the complete model.

In order to evaluate edge effects on various top-down control agents, we compared each mortality cause across habitat types using GLM models. For this, gall mortaliy (frequency scores of dead galls) due to each of the five mortality causes (ectoparasitoid, endoparasitoid, predation, unknown, fungus and total) was employed as response variable, while habitat type served as explanatory variable. To account for inadequate dispersion of the error distribution, we built GLMs using the binomial or quasibinomial family.

In addition, we adopted more general GLMs to identify key drivers of gall density considering two subsets of variables that combine habitat traits and biotic factors–a bottom-up-related one and another one with top-down focus. In both cases gall density in the understorey was assigned as response variable: In the first model we used vegetation density, dominance of *F*. *sylvatica*, canopy closure, habitat type and altitude as explanatory variables. In the second model we used parasitism rate (sum of endoparasitsm and ectoparasitsm rate), total mortality rate (sum of all mortality groups), canopy and altitude as explanatory variables. The minimal model was constructed by removing nonsignificant explanatory variables (p > 0.05) from the complete model. The best minimal model was selected using the adjusted R^2^.

Since parasitism rate (sum of endoparasitsm and ectoparasitsm rate) turned out to have a significant influence on gall density in the understorey, a final GLM was established to assess possible effects of the explanatory variables (canopy closure, habitat type, and altitude) on parasitism rate as response variable. Construction of the minimal model was conducted as described above. All models were submitted to residual analysis, so as to evaluate adequacy of error distribution. All analyses were conducted using the procedure”glm” in the software R (R Development Core Team, 2014).

## Results

### Edge effect on gall abundance

**Fig 2:**
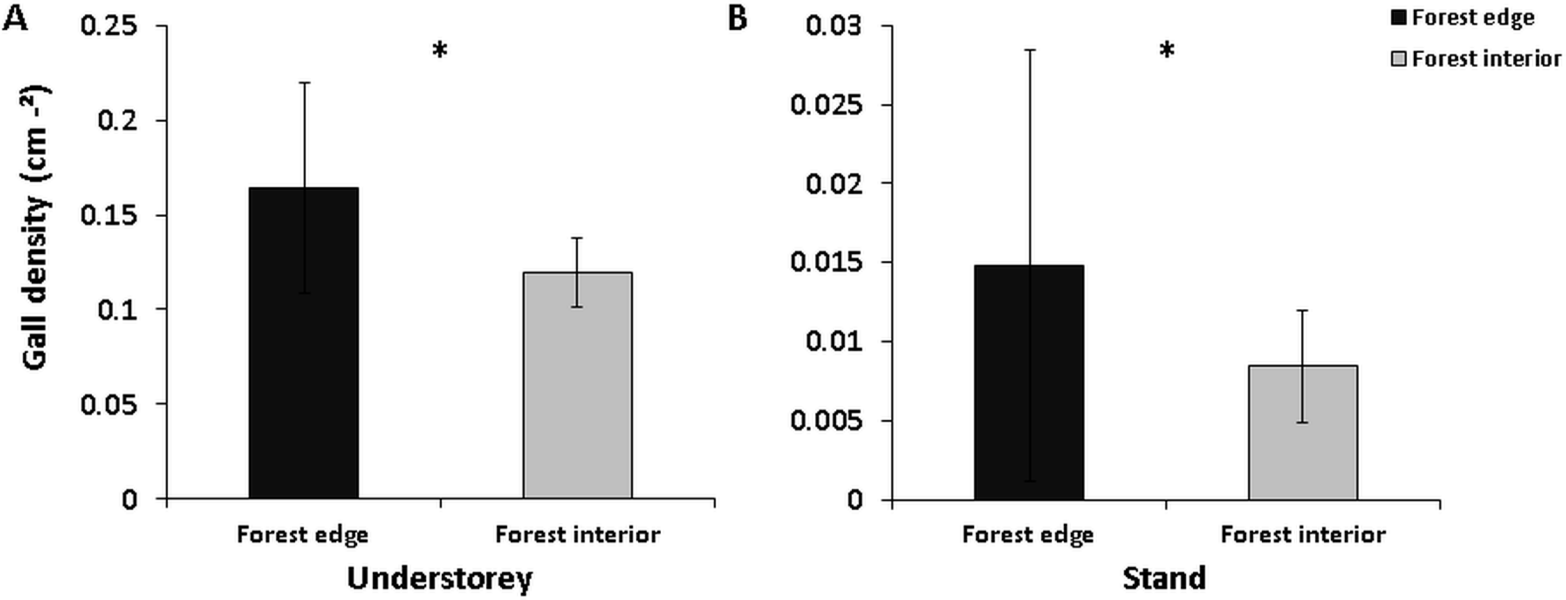
Edge effect on gall abundance of beech gall midge (*Mikiola fagi)*. Edge effect on gall abundance (density per cm² leaf area; mean ± standard deviation) in the understorey (left) and at stand level (right) of beech forests in the Northern Palatinate Highlands. Sample size (n) was 12 plots in the understory and 21 trees at stand level (Note: Different y-axis scales are used for legibility). Asterix indicates significant differences (α = 0.05) between habitat types.

In total we surveyed 299 trees in the understorey and 41 trees at stand level. The density of *Mikiola fagi* galls on beech foliage ranged from 0.008 ± 0.004 cm^-2^ at stand level (Fig 2, Stand) to 0.16 ± 0.05 cm^-2^ in the understorey (Fig 2, Understorey). In the edge-influenced understorey a typical beech leaf (ca. 15.82 cm^2^) showed a significant 30% increase in gall density compared to the forest interior (GLM: Df = 22; F = 7.15; Deviance = 0.037; p = 0.014). Despite lower overall gall density a similar strong edge effect was observed at stand level (GLM: Df = 39; F = 4.14; Deviance = 0.004; p = 0.049). To explore the factors driving gall density in each habitat type, we examined aspects such as bottom-up and top-down control as well as the role of edge-induced habitat traits. All following analyses refer to the understorey stratum, whereas Fig 5 refers to the stand level.

### The role of resource concentration

**Fig 3:**
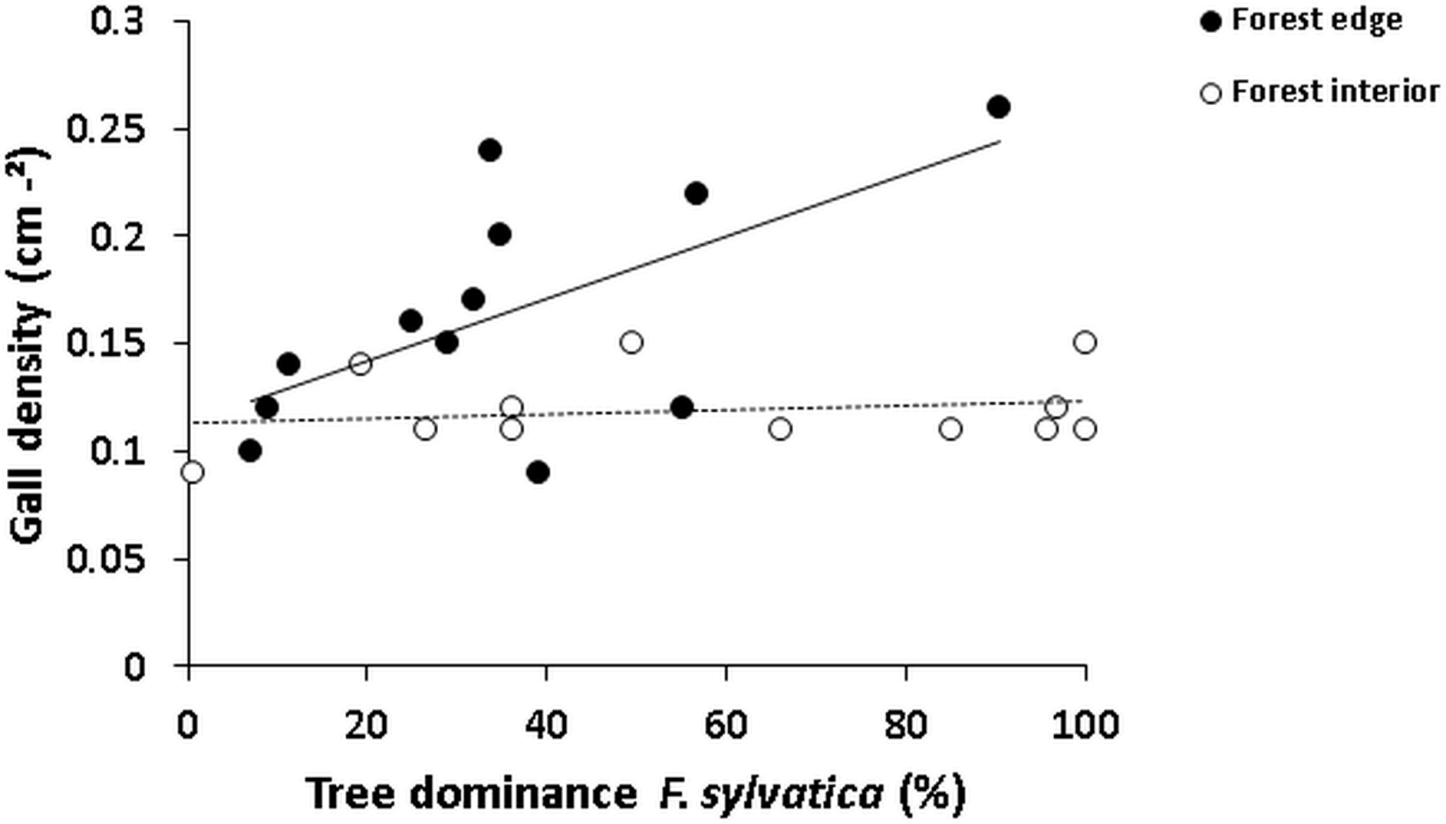
Relationship between gall density and resource availability for beech gall midge (*Mikiola Fagi*). Gall density (per cm² leaf area) and resource availability (relative beech dominance) along edge and interior plots (n = 12) in beech forests of the Northern Palatinate Highlands.

While interior plots often consisted of nearly pure beech stands, the dominance of *F*. *sylvatica* was reduced along the forest edge. Yet, when examining the importance of resource availability as a bottom-up factor for *M*. *fagi*, the dominance of *F*. *sylvatica* turned out to be a relative poor predictor for gall abundance (Table 1 (Model 1) and Fig 3). A significant interaction term indicated, however, that the effect of resource availability on gall abundance depended upon the variable habitat type (Fig 3): while at the forest edge gall abundance was clearly driven by resource availability, this effect was of minor importance in the forest interior.

**Table 1:** Effects of resource availability (tree dominance), parasitism rate and several habitat traits on the abundance of *Mikiola fagi* in the forest understorey of the Northern Palatinate Highlands.

Model	Response variable	Explanatory variable(s)	Distribution	df	F-statistics	p-value	Deviance	p-value (whole model)	adjusted R² (%)
**1**	**Gall density**		Gaussian				0.025	**0.002**	50
		Tree dominance *F*. *sylvatica* (%)		1	0.49	0.49			
		Habitat		1	13.69	**0.001**			
		Habitat x Tree dominance		1	6.36	**0.02**			
**2**	**Gall density**		Gaussian				0.028	**0.0001**	58
		Canopy closure (%)		1	17.50	**0.0004**			
		Altitude		1	11.00	**0.003**			
**3**	**Gall density**		Gaussian				0.029	**0.0005**	58
		Parasitism (%)		1	10.36	**0.004**			
		Canopy closure (%)		1	7.08	**0.015**			
		Altitude (m)		1	10.63	**0.004**			
**4**	**Parasitism**		Quasibinomial						
		Canopy closure (%)		1	20.10	-	1.21	**0.0002**	46

The minimal adequate generalized linear models (GLMs) showing the effects of resource availability (Model 1), parasitism rate (Model 3), and several habitat traits (Model 2 & 3) on the abundance of *Mikiola fagi* in the forest understorey of the Northern Palatinate Highlands. In addition, the effect of canopy closure on parasitism rate is displayed in the last row (Model 4).

### Top-down control on *M*. *fagi* galls

**Fig 4:**
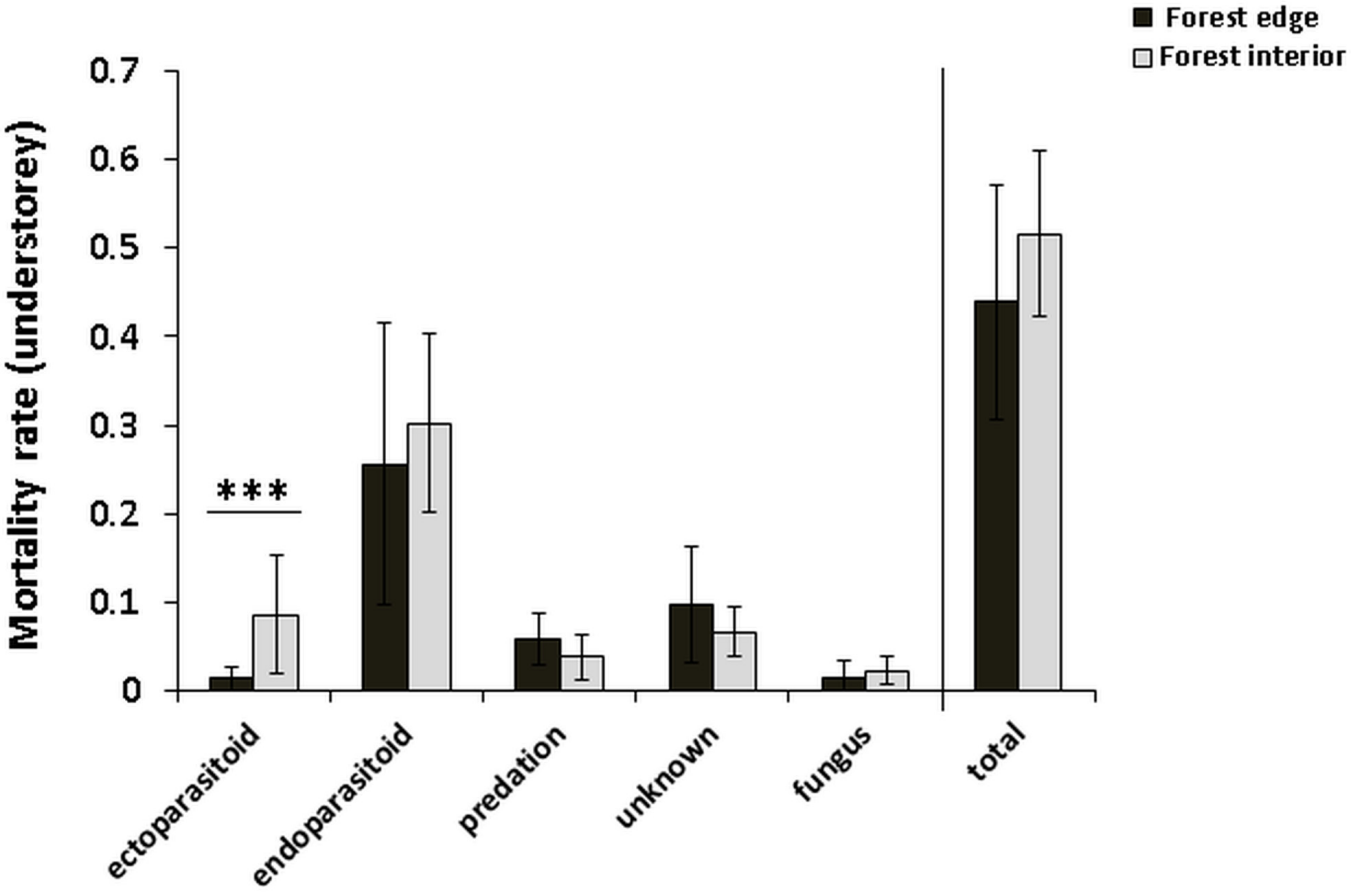
Edge effects on mortality rates of *Mikiola fagi* in the understorey. Data refers to the understorey of beech trees (*Fagus sylvatica*) in the Northern Palatinate Highlands. Means and standard deviation are indicated for different mortality causes as well as for total mortality rate (including all mortality groups). Significant differences between habitat types (α = 0.05) are indicated by asterix. See Table 2 for statistical details.

**Fig 5:**
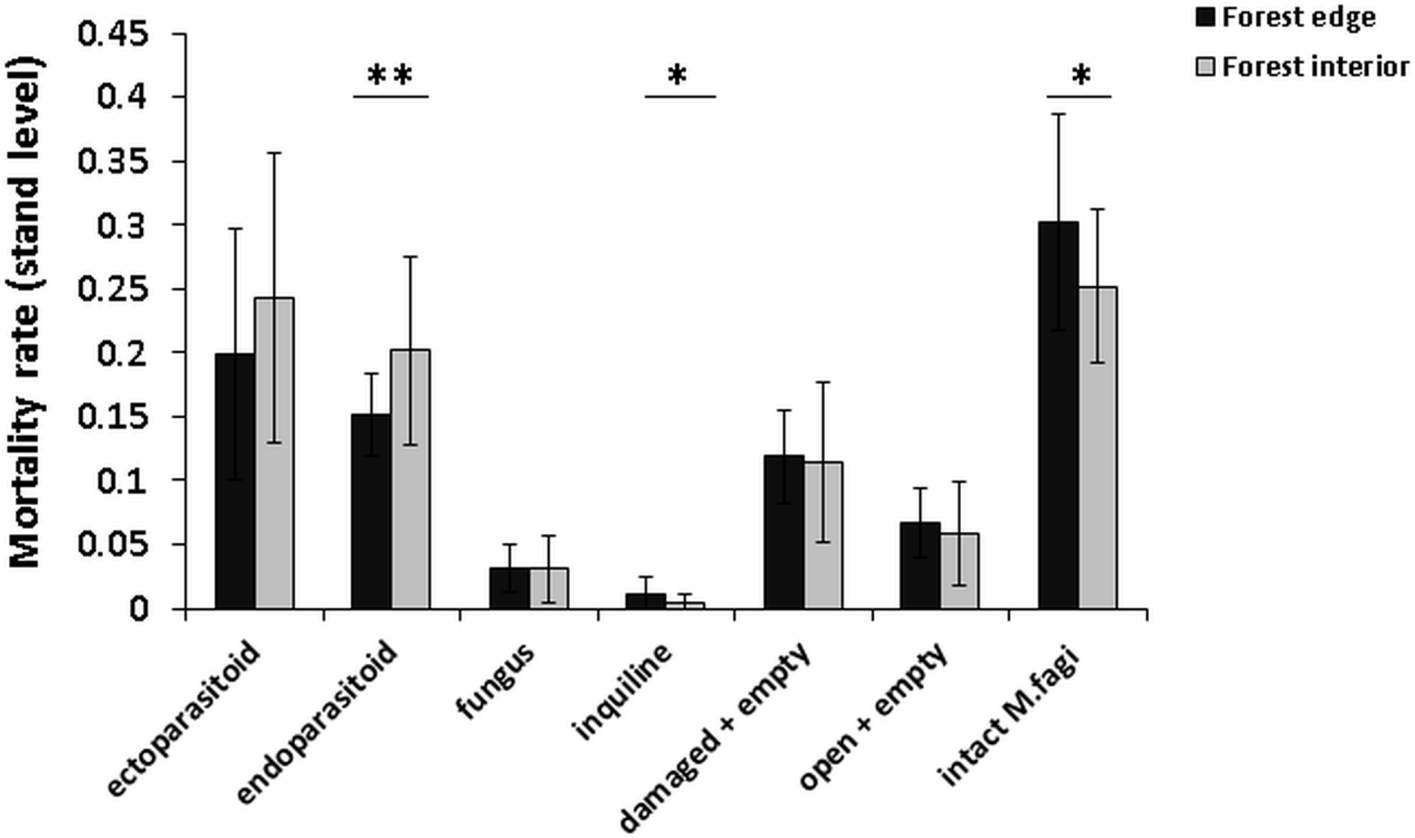
Edge effects on mortality rates of *Mikiola fagi* at stand level. Data refers to stand level of beech trees (*Fagus sylvatica*) in the Northern Palatinate Highlands. Means and standard deviations are given for different gall contents reflecting mortality causes as well as for intact *M*. *fagi* galls (last x-axis category). Asterix denote significant differences (α = 0.05) between habitat types. See Table 2 for statistical details.

The total mortality rate (sum of all mortality groups) of *M*. *fagi* varied from 2.5 to 10% and was not significantly influenced by the forest habitat (Table 2 and Fig 4). However, *M*. *fagi* death by ectoparasitoids was highly significantly affected by habitat, showing a reduced rate along the forest edge, while the other mortality rates remained unchanged at the forest edge (Table 2 and Fig 4). The largest proportion of *M*. *fagi* mortality was attributable to endoparasitoids (Fig 4) and, overall, the total parasitism rate (sum of endo- and ectoparasitoids in the understorey), suggests a considerable edge influence on *M*. *fagi* mortality by the parasitoid complex (0.39 in the forest interior versus 0.27 along the forest edge). Comparing these mean values with the three remaing mortality causes indicated that total parasitism rate might be the most important top-down factor on *M*. *fagi* and a key to explain higher survival at the forest edge. In fact, the stand-level examination of the leaf litter at the end of the season showed more galls with endoparasitoids in the forest interior habitat, and more galls with intact larvae in the edge habitat (Fig 5).

**Table 2:** Forest edge effects on various mortality causes of *Mikiola fagi* galls in the beech forest of the Northern Palatinate Highlands.

Level	Mortality causes	Distribution	F-statistics	Deviance	p-value
Understory (n = 24)		Quasibinomial			
	Ectoparasitoids		21.6	0.73	**0.0001**
	Endoprasitoids		0.72	2.05	0.41
	Predation		3.49	0.35	0.075
	Unknown		2.34	0.72	0.14
	Fungus		1.17	0.38	0.29
	Total		2.66	0.14	0.12
Stand (n = 42)		Quasipoisson			
	Ectoparasitoids		1.77	2.06	0.19
	Endoparasitoids		8.99	0.66	**0.005**
	Fungus		0.03	0.76	0.86
	Inquiline		5.78	0.53	**0.02**
	Damaged + empty		0.11	0.85	0.75
	Open + empty		0.67	0.77	0.42
	Intact *M*. *fagi*		5.08	0.77	**0.03**

Generalized linear models (GLMs) regarding forest edge effects on various mortality causes of *Mikiola fagi* galls in the beech forest of the Northern Palatinate Highlands. While the first models (upper table section, cf. Fig 4) refers to mortality of galls on understory foliage during the vegetation period, the second models (cf. Fig 5) is based on stand level data collected from the leaf litter at the end of the vegetation season. Significant p-values are in bold.

### Main factors driving gall density

In an attempt to investigate variability in gall abundance in the understorey by involving all explantory variables at our disposal, our model selection revealed two adequate models, which both were able to explain a considerable proportion (58%) of variation in gall density (Table 1; Model 2 & 3). Both models included the fragmentation-related variables canopy closure and altitude, which were negatively correlated with gall abundance (see specification below). As structural parameter that reflects understorey light regime, canopy closure pointed to a positive relationship between gall density and light exposure. The effect of altitude and concomitant microclimatic changes suggest a positive relationship between gall abundance and warmer temperature. Although the model did not include habitat per se, it provided indirect information about the habitat types with negative coefficients for altitude (-0.0002) and canopy closure (-0.0075), indicating that the forest interior plots are generally associated with i) higher plot elevation and (ii) lower canopy openness than edge plots. The second model incorporated total parasitism rate in addition to canopy closure and altitude; it retained just about the same explanatory power (58%) and corroborated the negative relationship between top-down pressure and gall abundance.

**Fig 6:**
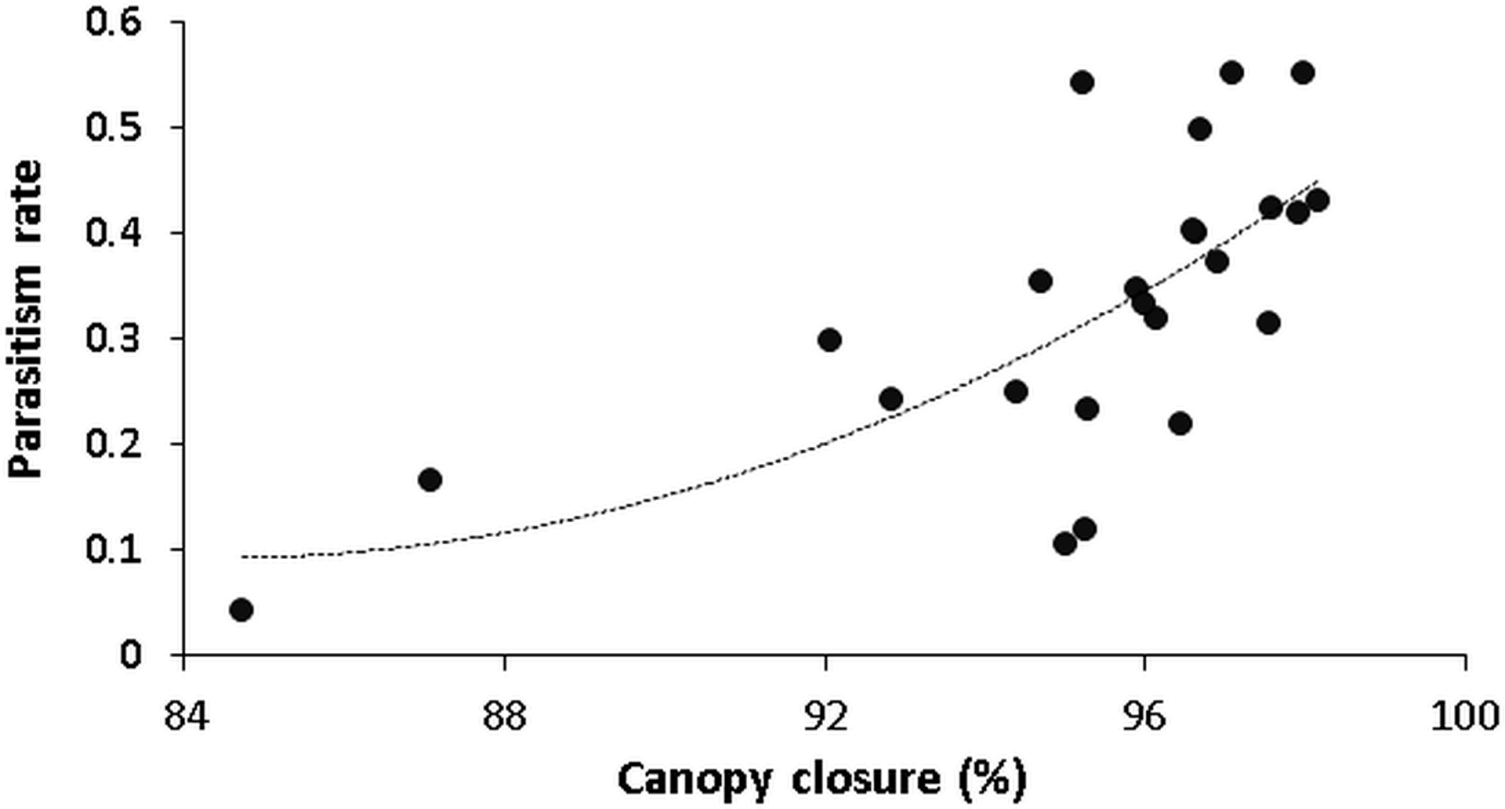
Relationship between parasitism rate in beech gall midge (*Mikiola fagi*) and canopy cover. Data refers to the understorey of beech trees (*Fagus sylvatica*) in the Northern Palatinate Highlands. The curve line is described by: y = (exp (-17.08 + 0.17 * x) / 1 + exp (-17.08 + 0.17 * x)).

Parasitism as top-down factor showed up to be the only “biotoc” feature explaining across-habitat variation in gall abundance. The fact that parasitism was significantly influenced by canopy closure (coefficient 0.17; Table 1 (Model 4) and Fig 6) provides some insight into the underlying mechanism behind the edge-induced release from top-down control (Fig 4): With an explanatory power of 46% canopy closure reflects a negative relationship between parasitism rate and light exposure.

## Discussion

**Fig 7:**
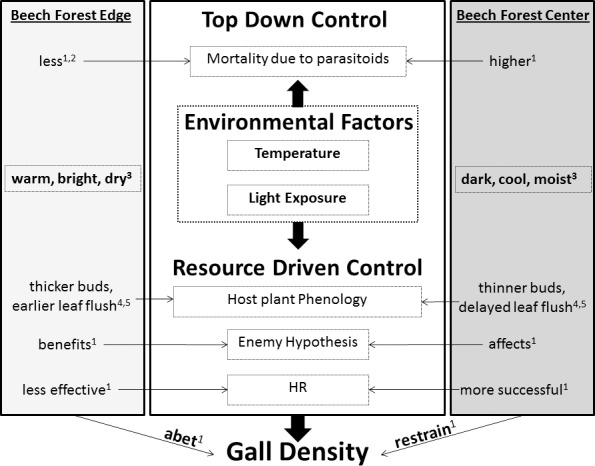
Potential mechanisms and habitat-driven modulators of *M*. *fagi* gall density across edge-affected beech forests. Bold black arrows indicate interactions between abiotic factors and trophic mechanisms as well as the overall impact on population control; thin arrows denote habitat-induced effects on the respective mechanisms; HR = Hypersensitivity reaction (sensu [24]). Superscripts refer to the following references: (1) this study, (2) [52], (3) [5], (4) [42], (5) [40].

To our knowledge, this is the first empirical study addressing, on a population level, the impact of forest edge on a widespread galling herbivore in temperate deciduous forest. It demonstrated a substantial edge effect on the abundance of the beech gall midge (*M*. *fagi*) and identified potential key factors driving gall density across fragmented forests. We found 30% higher gall abundance in the edge habitat as well as lower mortality rates due to decreased top-down control, particularly regarding parasitism pressure. While the hypothesized impact on trophic interactions was clearly supported as a mechanism to explain variation in gall density, our findings also emphasize the direct and indirect role of abiotic factors. In a conceptual model that synthesizes existing knowledge and insights from the present research, we summarize the key mechanisms and habitat-driven modulators of gall density across edge-affected beech forests (Fig 7).

### Edge-induced changes of environmental factors and potential interrelations with bottom-up factors

This investigation identified two sources of variation in the density of *M*. *fagi* galls in beech forests. On one hand it confirms observations by Skuhravy and Skuhrava [21] indicating a vertical stratification with highest abundance in the lower parts of the tree crowns (but see contrasting evidence in [25]). On the other hand, it represents the first record of forest edge-induced proliferation of *M*. *fagi*. One of the most robust GLM models provided high explanatory power of the habitat traits canopy closure and altitude. Both factors are related to microclimatic conditions such as light exposure, temperature and humidity. According to the harsh environment hypothesis, gall inducing insects are generally associated with harsh ecophysiological conditions [34,36,38,39]. In fact, studies on leaf parameters in the same study plots demonstrated the more xeromorphic nature (e.g., lower specific leaf area and lower water content) of beech trees at the edge compared to the forest interior (Kelch N. unpublished diploma thesis).

In our study region, altitude seemed to be an attribute of the interior forests (which escaped historical fragmentation by being situated on agriculturally less valuable hilltops) and was negatively correlated with gall abundance. The observed decrease in gall abundance with increasing altitude is plausibly explained by climatic changes such as the decline in temperature. Indeed, Urban [40] described *M*. *fagi* to ovipost 2.5 times more often on early budding beeches, and temperature has a strong effect on the average day of budburst in beech [41]. This is supported by the fact that, with increasing altitude, the vegetation period of *F*. *sylvatica* shifts to later dates [42]. Moreover, temperature changes may also directly influence the performance of galling insects [43]. The importance of close synchrony between bud burst and adult emergence or egg hatch for many gall-inducing insects, particulary those that ovipost before leaf flush, is predicted by the phenological synchrony hypothesis [44] (Fig 7: Host plant phenology). Microclimatic preferences of galling insects are often considered a key factor determining the location of oviposition. In our case study, warmer microclimatic conditions in lower altitudes along forest edges (naturally hotter) probably influenced the host-phenology of *F*. *sylvatica*, not only concerning the timing of budbreak, but also the development of the buds themselves. In beech trees, the light regime in early August determines the sun versus shade leaf characteristics of the following season: Sun-leaf primordia show five, and shade-leaf primordia four layers of mesophyll meristem cells [45], making sun-buds thicker and thus preferred over shade-buds by *M*. *fagi* [40] (Fig 7: Host plant phenology). Such increased oviposition in sun-exposed habitats of forest edges has also been observed in other insects, such as butterflies [46].

Despite the exclusive host specificity of *M*. *fagi* and contrary to published cases of edge-induced proliferation of tropical cecidomyiid gallers [47] (and other insect herbivores reviewed in [8]), our findings of reduced gall abundance in dense beech stands of the forest interior did not conform with the hypothesis of ressource concentration (sensu [35]). This is in line with Fernandes et al. [24], who found leaf density to be a poor predictor of *M*. *fagi* abundance within beech trees. In scenarios with almost no resource limitation, such as the interior of quasi-monodominant, managed beech forests, the resource concentration hypothesis may therefore be of minor relevance. In fact, the strong correlation between gall density and beech dominance in the species-rich forest edge habitat lends support to this interpretation.

Another indirect benefit to *M*. *fagi* may result from edge-induced changes in the ecophysiological response of beech trees. Following this line of thought, the spatial distribution pattern of gall density may also arise from bottom-up based processes other than resource availability. In galling insects, plant resistance mechanisms play a major role in determining failure and success of the herbivore. More particularly, in beech the so-called hypersensitive reaction (HR) represents an important factor acting against galling populations [24]. This induced defense reaction affects key phases of gall induction and development: A chemical shock and osmotic change caused by larval saliva injected into the plant tissue is the first step in gall induction, and its modality determines the process of plant defense or gall development (reviewed in [48]). If that shock is low in intensity, the plant will respond with gall development, whereas high intensity leads to cell apoptosis followed by a rejection of the inducing insect, i.e., a HR response. Keeping in mind that sun leaves of beech at the forest edge have a relative high osmotic pressure [49], the osmotic shock should be relatively low, resulting in a successful gall induction due to compromised plant defense. On the other hand, osmotic differences between shade leaves in the forest interior and the larval saliva is rather high and may thus promote the HR (Fig 7: HR). While this causal path offers a plausible explanation, it remains hypothetical at this stage due to the lack of empirical data. Nevertheless, such bottom-up-mediated influence on gall development via edge effects on HR opens a promising research perspective for elucidating the mechanisms driving gall proliferation in fragmented forest landscapes. In fact, we believe that *M*. *fagi* and beech trees may represent an excellent model system to test this hypothesis by addressing plant defense directly in springtime, when HR occurs.

### Top-down factors

The present findings are consistent with earlier studies emphasizing a distinct parasitoid complex as prevalent top-down factor controlling beech gall midge populations [20]. In addition, our results provide novel evidence that both ecto- and endoparasitism rates of *M*. *fagi* are considerably and negatively affected by forest edge, suggesting that edge-associated gall abundance is a case of ecological release from natural enemies. While the increased susceptibility of the third trophic level to habitat fragmentation (i.e., the trophic-level hypothesis sensu Holt [29]) has frequently been reported to explain such herbivore release [50,51], negative edge-influence on top-down processes are less well documented [8]. To give an example, the hyperabundance of leaf-cutting ants along tropical forest edge zones has been attributed to the edge-mediated reduction of parasitism pressure by moisture-loving phorid flies [52]. Our findings of the reverse relation between light exposure and parasitism rate support the idea that environmental edge conditions are responsible for reduced parasitism (Fig 7: Mortality due to parasitoids). Additionaly, although not addressed here, the parasitoid community itself could also suffer from a higher top-down pressure at the forest edge, e.g. via predation by spiders, as suggested by Wimp et al. [53]. Finally, limited parasitoid success might be mediated by edge-induced changes in the host plant phenology (Fig 7: Enemy hypothesis) as followed from an observation by Urban [40], that galls on early budding beeches (corresponding to edge trees; see above) grow bigger and are characterized by a thicker gall wall, which may protect the larvae from parasitism [54,55]. While we lack empirical verification in the *M*. *fagi* system, such alteration of trophic interactions via light-induced shifts in gall morphology plausibly supports the idea of multiple interrelated mechanisms behind the response of beech gall midge to edge-affected beech forests (Fig 7).

## Conclusions

Synthesizing the above analysis, we can conclude that forest edge proliferation of gall-forming *M*. *fagi* larvae is caused by a complex interplay of abiotic changes and trophic control mechanisms (Fig 7). Most prominently, we found strong evidence indicating that the microclimatic regime of fragmented/edge-influenced beech forests acts in synergistic concert with top-down pressure by ecto- and endoparasitoids, turning *M*. *fagi* into a winner species (sensu [56]) in fragmented temperate beech forests. Our findings generally corroborate published evidence on community level, indicating that forest edges benefit galling insects in terms of species richness and abundance [17, 18], but go beyond these by exploring the mechanisms driving the patterns on population level. To give an example, our results are partly conform with the findings of Altamirano et al. [57] that galling insects directly benefit from abiotic edge conditions, but they clearly extend the underlying mechanisms to trophically mediated effects. Interestingly, *M*. *fagi* as a highly specialized herbivore represents an exception from the specialization hypothesis [29, 50, 58], predicting that specialists are more sensitive to edge formation. The reasons for this warrant further investigation, since similar edge responses are typically documented for generalist herbivores [8].

Furthermore, the drastic edge-related increase (30%) in gall density may have important implications in the contexts of silviculture and conservation. In fact, galls act as strong sinks in the plant´s physiology [59] and a high gall abundance implies negative consequences for the host plant [44]. However, while *M*. *fagi* is known to cause a remarkable loss of assimilation area/photosynthetic productivity in *F*. *sylvatica* trees [21] and Kelch N. (unpublished diploma thesis), it´s economic and conservation relevance seems widely neglected (pers. comm. F. Engels, German Research Institute for Forest Ecology and Forestry, Trippstadt). Therefore, in view of (i) the increasing proportion of edge habitats [3] and (ii) the documented benefits of *M*. *fagi* from edge microclimatic regimes that are likely to be enhanced by future climate change [60], we call for further investigations exploring both the pest status of this galling insect and the modulators of its biological control.

## Supporting Information

S1 Table Minimal data set for forest interior and forest edge plots in the Northern Palatinate Highlands. (DOC)
