# Supplementary material for: Mechanisms Driving Galling Success in a Fragmented Landscape: Synergy of Habitat and Top-Down Factors along Temperate Forest Edges
Source: PLoS One. 2016 Jun 16;11(6):e0157448. doi: 10.1371/journal.pone.0157448 (PMC4910982; doi:10.1371/journal.pone.0157448)
Supplement: S1 Table — (DOC) [file pone.0157448.s001.doc]

**S1** **Table. Minimal data set for forest interior and forest edge plots in the Northern Palatinate Highlands.**

| Habitat | GPS-Data | Age (y) | Gall density [cm -²] (understorey) | Gall density [cm -²] (stand level) | ASL | Canopy closure [%] | Parasitism rate (understorey) | Parasitism rate (stand level) | Total mortality (stand level) |
| --- | --- | --- | --- | --- | --- | --- | --- | --- | --- |
| Edge | N49 34.394 / E7 35.921 | 78 | 0.11 | / | 427 | 96.45 | 0.22 | / | / |
| Edge | N49 34.134 / E7 35.581 | 79 | 0.17 | / | 449 | 94.70 | 0.35 | / | / |
| Edge | N49 34.021/ E 7 35. 831 | 73 | 0.15 | 0.1 | 433 | 95.89 | 0.35 | 0.47 | 0.76 |
| Edge | N49 33.763 / E7 35.213 | 129 | 0.09 | 0.007 | 425 | 92.84 | 0.24 | 0.32 | 0.62 |
| Edge | N49 33.509 / E7 35.299 | 69 | 0.12 | 0.008 | 433 | 96.00 | 0.33 | 0.36 | 0.71 |
| Edge | N49 35.320 / E7 53. 133 | 54 | 0.24 | 0.04 | 285 | 95.26 | 0.12 | 0.30 | 0.60 |
| Edge | N49 35.472 / E7 52.035 | 46 | 0.22 | / | 383 | 84.73 | 0.04 | / | / |
| Edge | N49 36. 152/ E7 50. 740 | 119 | 0.12 | / | 383 | 95.01 | 0.10 | / | / |
| Edge | N49 35.966 / E7 56.403 | 84 | 0.2 | / | 389 | 95.29 | 0.23 | / | / |
| Edge | N49 36.585 / E7 56.893 | 37 | 0.14 | / | 315 | 97.09 | 0.55 | / | / |
| Edge | N49 40.502 / E7 53.785 | 92 | 0.26 | 0.02 | 358 | 87.08 | 0.16 | 0.25 | 0.70 |
| Edge | N49 41.387 / E7 54.036 | 98 | 0.16 | / | 337 | 95.22 | 0.54 | / | / |
| Interior | N49 34.330 / E7 35.326 | 71 | 0.11 | / | 524 | 97.60 | 0.42 | / | / |
| Interior | N49 34.465 / E7 34.704 | 143 | 0.11 | 0.007 | 590 | 96.64 | 0.40 | 0.53 | 0.80 |
| Interior | N49 34.663 / E7 34.826 | 123 | 0.11 | 0.004 | 559 | 96.62 | 0.40 | 0.44 | 0.77 |
| Interior | N49 34.523 / E7 35.010 | 63 | 0.11 | 0.007 | 563 | 94.41 | 0.25 | 0.32 | 0.67 |
| Interior | N49 35.198 / E7 34.233 | 97 | 0.12 | / | 438 | 98.16 | 0.43 | / | / |
| Interior | N49 36.988 / E7 55.952 | 62 | 0.11 | / | 606 | 96.17 | 0.32 | / | / |
| Interior | N49 37.676 / E7 54.911 | 60 | 0.11 | 0.04 | 640 | 92.08 | 0.30 | 0.62 | 0.96 |
| Interior | N49 36.271 / E7 53.606 | 74 | 0.14 | / | 573 | 96.92 | 0.37 | / | / |
| Interior | N49 39.114 / E7 57.860 | 52 | 0.15 | / | 364 | 96.69 | 0.50 | / | / |
| Interior | N49 39.207 / E7 56.813 | 115 | 0.09 | / | 395 | 97.99 | 0.55 | / | / |
| Interior | N49 41.632 / E7 56.894 | 60 | 0.14 | / | 358 | 97.57 | 0.31 | / | / |
| Interior | N49 39.171 / E7 54.975 | 126 | 0.12 | 0.007 | 491 | 97.92 | 0.42 | 0.46 | 0.83 |
